# Reducing Over-Utilization of Cardiac Telemetry with Pop-Ups in an Electronic Medical Record System

**DOI:** 10.7759/cureus.1282

**Published:** 2017-05-29

**Authors:** Wajeeha Rizvi, Cyrus M Munguti, Jeet Mehta, K. James Kallail, Darrell Youngman, Samer Antonios

**Affiliations:** 1 Internal Medicine, University of Kansas School of Medicine - Wichita; 2 Cardiology/internal Medicine, Via Christi Hospitals Wichita

**Keywords:** telemetry overutilization, cardiac telemetry, electronic medical record, reducing telemetry overuse

## Abstract

Non-invasive cardiac monitoring has well-established indications and protocols. Telemetry is often overused leading to a shortage of tele-beds and an increment of hospital expenses. In some cases, patients are kept on telemetry longer than the indicated length because providers are unaware of its ongoing use. We investigated the effect of reminder pop-ups, incorporated into an electronic medical record (EMR), on minimizing the use of telemetry. Three regional hospitals implemented an electronic pop-up reminder for discontinuing the use of telemetry when no longer indicated. A retrospective analysis of data for patients on telemetry, outside of the intensive care unit (ICU), was conducted and comparisons were drawn from pre- and post-implementation periods. A composite analysis of the number of days on telemetry was calculated using the Kruskal-Wallis test. With the implementation of the pop-up reminder, the median number of days on telemetry was significantly lower in 2016 than in 2015 (2.25 vs 3.61 days, p < 0.0001). Overutilization of telemetry is widely recognized, despite not being warranted in non-ICU hospitalizations. The implementation of a pop-up reminder built into the electronic medical record system reduced the overuse of telemetry by 37% between the two time periods studied.

## Introduction

Cardiovascular disease causes significant morbidity and mortality in the United States [1]. Prompt recognition and treatment of arrhythmias among admitted patients is one way of reducing this burden. The American Heart Association (AHA) has published guidelines on the use of cardiac telemetry among non-intensive care unit (ICU) patients; however, it is often difficult to translate the guidelines into practice [2]. Furthermore, the overuse of telemetry can often outweigh the benefits, causing harm to patients and increasing healthcare costs [2-3]. Continuous healthcare reformation warrants efficient and cost-effective health care practices. The overuse of telemetry often leads to a shortage of tele-beds and an increment of expenses [4]. The American Board of Internal Medicine (ABIM) does not recommend the use of telemetry monitoring outside the ICU without a continuation protocol [5]. As of 2009, a day’s cost of telemetric monitoring was at least $1,400 per patient. In some cases, patients are kept on telemetry longer than indicated because providers are unaware of its ongoing use [6-7]. Several strategies have been studied to reduce the overuse of cardiac telemetry [6,8]. While there is a lack of consensus on the most effective method to improve the overutilization of cardiac telemetry [9], we developed a continuous quality improvement strategy and investigated the effect of reminder pop-ups, in an electronic medical record (EMR) system, on the duration and utilization of cardiac telemetry.

## Materials and methods

Three regional hospitals in Wichita, Kansas, (using the same EMR system) implemented an electronic pop-up for discontinuing the use of telemetry when no longer indicated. The criteria for discontinuing the use of telemetry included the appearance of a pop-up after 48 hours of the patient being on telemetry. The pop-up would alert the clinician to either continue telemetry or discontinue it if no longer required. The study design was submitted to the Institutional Review Board (IRB) in Wichita. It was determined that the study did not constitute human subjects research; therefore, IRB approval was not required. The board issued a waiver letter and all ethical guidelines were followed.

A retrospective analysis of data for patients on telemetry, outside of the ICU, was conducted during the period of April 2015 to October 2015 (pre-implementation) and seven months after the placement of the pop-ups (post-implementation) between April 2016 to November 2016. During this period, a total of 34,572 patient hospitalizations were reviewed.

## Results

**Figure 1:**
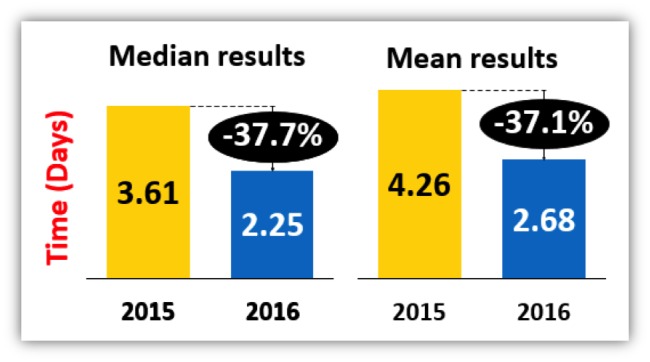
Impact of pop-up reminders on mean and median number of days on cardiac telemetry

The median number of days on telemetry (pre-implementation period) was 3.61. This was reduced to 2.25 days following the implementation of electronic record pop-ups. This reduction was statistically significant (2.25 days vs 3.61 days, p < 0.0001. (Table 1) (Figure 1). The mean number of days on telemetry was 4.26 and 2.68 in 2015 and 2016, respectively.

 

**Table 1:** Comparison of the mean number of days on telemetry, pre- and post-implementation of electronic pop-ups

Year	N	Mean Number of Days on Telemetry (SD)	Median Number of Days on Telemetry (IQR*)	Minimum, Maximum Days on Telemetry	First Quartile, Third Quartile	P value
2015	14,192	4.26 (4.08)	3.61 (3.97)	0, 80.92	1.57, 5.54	< 0.0001
2016	20,380	2.68 (1.98)	2.25 (1.74)	0, 30	1.71, 3.45	

 

## Discussion

The implementation of a pop-up reminder built into the EMR system reduced telemetry overuse at our institution by 37% between the two time periods studied. Cardiac telemetry use has been expanding exponentially for the past 30 years. Initially, it was recommended for cardiac and sometimes non-cardiac patients in the ICU [10]. With time, its use had advanced to patients in non-ICU settings [8]. Overutilization of telemetry is recognized widely, despite not being warranted in non-ICU hospitalizations.

Guidelines for in-hospital cardiac monitoring have been published by the American College of Cardiology [11]. However, cardiac telemetry continues to be overused due to non-adherence to the guidelines by physicians or due to unawareness of telemetry continuation days [6-7,10]. The number of days on telemetry is a clear component in the increment of hospital expenses. The ABIM does not recommend the use of telemetry outside of the ICU setting without a continuation protocol [5]. Clinician education is critical to understand the risks and benefits of the use of telemetry in non-ICU patients. Furthermore, patient satisfaction has been shown to increase due to the decreased number of alerts and cardiac alarms [2].

## Conclusions

The implementation of electronic pop-up reminders reduced the duration of telemetry in non-ICU settings. Being a time bound analysis, we were unable to show if this change can persist beyond the project, or ascertain the effect of this project on individual physician practices on discontinuation of telemetry. The financial savings from early telemetry discontinuation is inferred and further research would ascertain this.
